# Novel antibody competition binding assay identifies distinct serological profiles associated with protection

**DOI:** 10.3389/fimmu.2023.1303446

**Published:** 2023-12-11

**Authors:** Jessica S. Bolton, Randall S. MacGill, Emily Locke, Jason A. Regules, Elke S. Bergmann-Leitner

**Affiliations:** ^1^ Biologics Research & Development, Walter Reed Army Institute of Research (WRAIR), Silver Spring, MD, United States; ^2^ Center for Vaccine Innovation and Access, PATH, Washington, DC, United States

**Keywords:** serology, circumsporozoite protein, antigen-specificity, equivalency, vaccine, protection, malaria

## Abstract

**Introduction:**

Pre-erythrocytic malaria vaccines hold the promise of inducing sterile protection thereby preventing the morbidity and mortality associated with *Plasmodium* infection. The main surface antigen of *P. falciparum* sporozoites, i.e., the circumsporozoite protein (CSP), has been extensively explored as a target of such vaccines with significant success in recent years. Systematic adjuvant selection, refinements of the immunization regimen, and physical properties of the antigen may all contribute to the potential of increasing the efficacy of CSP-based vaccines. Protection appears to be dependent in large part on CSP antibodies. However due to a knowledge gap related to the exact correlates of immunity, there is a critical need to improve our ability to down select candidates preclinically before entering clinical trials including with controlled human malaria infections (CHMI).

**Methods:**

We developed a novel multiplex competition assay based on well-characterized monoclonal antibodies (mAbs) that target crucial epitopes across the CSP molecule. This new tool assesses both, quality and epitope-specific concentrations of vaccine-induced antibodies by measuring their equivalency with a panel of well-characterized, CSP-epitope-specific mAbs.

**Results:**

Applying this method to RTS,S-immune sera from a CHMI trial demonstrated a quantitative epitope-specificity profile of antibody responses that can differentiate between protected vs. nonprotected individuals. Aligning vaccine efficacy with quantitation of the epitope fine specificity results of this equivalency assay reveals the importance of epitope specificity.

**Discussion:**

The newly developed serological equivalence assay will inform future vaccine design and possibly even adjuvant selection. This methodology can be adapted to other antigens and disease models, when a panel of relevant mAbs exists, and could offer a unique tool for comparing and down-selecting vaccine formulations.

## Introduction

Understanding immune correlates of protection can greatly facilitate the development of effective vaccines. Antibodies have been identified as correlates of protection for some vaccines [reviewed in (1)]. The *Plasmodium falciparum* sporozoite’s Circumsporozoite Protein (CSP) is the target of most pre-erythrocytic malaria vaccines. This includes the vaccine RTS,S/AS01_E_ that is based on a CSP fragment, and the vaccine was recommended for widespread use among children in sub-Saharan Africa and in other regions with moderate to high *P. falciparum* malaria transmission. This recommendation was based on results from an ongoing pilot implementation program in more than 900,000 children in Ghana, Kenya and Malawi (2–5). In 2022, RTS,S/AS01_E_ (Mosquirix) became the first malaria vaccine to receive prequalification approval from the WHO, which enables UNICEF to purchase the vaccine (6).

Additional CSP-based vaccine candidates have also shown promise in clinical studies, thus bringing the community closer to additional protective malaria vaccine(s) [(7), and Robben et al., manuscript in preparation]. Assessing vaccine efficacy through surrogate immunological parameters is hampered by the lack of confirmed immune correlates of protection mediated by CSP-specific immune responses. Antibody titers, as measured by enzyme linked immunosorbent assays (ELISA), alone have not been able to predict protection (8–10), requiring a multi-faceted assessment of antibody isotypes, functional activity, and avidity (9, 11). Various rodent models have been employed to assess and screen candidate vaccines, and some are undergoing interrogation for correlation with controlled human malaria infection model (CHMI) results (Locke et al., manuscript in preparation). However, the CHMI trial continues to be the best way to assess vaccine efficacy in early, reasonably sized clinical studies, therefore lengthy development time and great cost must be expended before a definitive clinical field efficacy readout can be obtained. The objective of the present study was to develop a serological competition assay, the CSP-based assay for serological quantification and equivalency (CBASQE), that assesses the equivalency of vaccine-induced antibodies in relation to well-characterized monoclonal antibodies against key epitopes of CSP. In this assay, the equivalence, i.e., the ability of CSP vaccine-induced antibodies to successfully compete with well characterized, relevant mAbs for binding to their epitopes, determines epitope specificity, implied avidity, and concentration of antibodies in sera from vaccinees. One of the key features of the assay – and difference from standard assays for evaluating serum samples - is its ability to evaluate both preclinical and clinical samples, unbiased by the use of different, species-specific secondary antibodies (Bolton et al. manuscript in preparation).

Our CBASQE assay was developed using an electro-chemiluminescence immune assay (ECLIA)-based multiplex platform that has unique qualifying features: (1) high sensitivity with exceptionally low inter- and intra-assay variability; (2) wide linear range over 4-5 logs; and (3) suitability for testing closely related antigens without cross reactivity due to antigenic similarity and competition; and (4) a proven suitability for samples from human malaria vaccinees (12–15).

The CBASQE assay was designed as a competition assay using well-defined monoclonal antibodies specific for crucial epitopes within CSP with most having demonstrated functional activity in preclinical models and/or the clinic. To this end, we selected the N-terminus specific mAb 5D5 (16, 17), the junctional region-specific (NPDP) mAb CIS43 (18, 19), the minor repeat-specific (NVDP) mAb L9 (18, 20, 21), the major repeat-specific (NPNA_6_) mAb 317 (19, 22–24), and the C-terminal mAbs 236 (16) and 369. These important epitopes are linear except for those in the C-terminus. Importantly, no steric inhibition between these mAbs, even with the two C-terminus-specific mAbs, is anticipated nor has it been observed (Bolton et al, manuscript in preparation) allowing for the independent quantitation of these regional responses. Plate antigen selection was based on the reported roles of the various regions of CSP during sporozoite migration to the liver and invasion of host cells: The N-terminus sequence plays a crucial role during the passage of the sporozoite to the liver (25, 26). CSP undergoes cleavage of the N-terminus right before infecting hepatocytes, a process during which the C-terminus is revealed, allowing it to bind to cellular receptors and mediate entry into the host cell (25). The junctional region and the repeat region are critical for successful invasion, at least for some of the *Plasmodium* species, particularly *P. falciparum*. The junction-specific mAb CIS43 and minor repeat-specific mAb L9 have recently been tested in clinical trials (NCT04206332 and NCT05019729) for their ability to protect against progression to blood-stage infections after intravenous delivery with subsequent sporozoite CHMI and demonstrated significant efficacy (27, 28). The major repeat region is represented by six NPNA repeats, which is long enough to measure binding of homodimerizing mAbs (19, 24, 29–32), but minimizes cross reactive binding by mAbs CIS43 and L9 (21, 33). The CBASQE assay was used to dissect repeat region-specific antibody responses to determine the importance of junction *vs*. minor- *vs*. major repeat-specific antibodies.

The plate antigens representing the CSP C-terminus used in our assay span most of the non-repeat region of the RTS,S vaccine construct (CSP aa 272-389) and harbor many highly polymorphic amino acid positions presumably selected by immune pressure present in the field. A subset of these sequences was observed to be associated with allele-specific vaccine protection in a previous study (34). The role of C-terminal antibodies is disputed, especially when assessing the functional activity of mAbs in rodent models (35), but there is mounting evidence from studies of clinical samples suggesting antibodies with C-terminal specificity are indeed a component of protective responses following RTS,S/AS01 vaccination (15, 36–39). Furthermore, previous work from our laboratory has demonstrated that the breadth of C-terminal antibody responses is associated with protection (15). In that study, eight variant C-terminal peptides derived from a database of naturally occurring CSP sequences in an RTS,S/AS01_E_ phase III ancillary genotyping study (34) were tested for reactivity with sera from malaria-naïve RTS,S vaccinees (15). Neafsey et al. had determined the Hamming distance of each of the variant peptides to the RTS,S vaccine’s 3D7 sequence at polymorphic amino acid positions (34). The results of this C-terminal breadth assay suggested the breadth of antibody reactivity is an indicator of a focused response against the conserved portion of the molecule. These responses to the conserved region are likely mediating functional activity. To account for variants present in the field, the assay described here includes the 3D7 sequence of the C-terminus that is represented in many CSP-based vaccines including RTS,S (40) and R21 (41). The H18 peptide represents mid-Hamming distant divergence [Hamming distance = 5], and the H50 represents the most divergent variant [Hamming distance = 10] from the 3D7 sequence.

To demonstrate the power of the novel CBASQE assay in discerning qualitative differences and assessing antibody equivalency, we tested a panel of clinical samples from an RTS,S trial (42) with known protective status of the vaccinees (15, 42). With these data, we established serological profiles of protective responses in humans induced by RTS,S vaccination and can assess potential contributing and hierarchical roles of the distinct epitopes in protection. These results emphasize the importance of dissecting minor/major repeat and C-terminal specific immune responses and their role in mediating protection. Systematic comparisons of CBASQE profiles of preclinical, Phase I/IIa and Phase IIb samples will be invaluable when assessing the contributions of region-specific antibodies to protection and guide future vaccine design by indicating the critical epitopes to be included in next-generation CSP-based vaccines designed to induce both high quantity and high-quality vaccine responses. Such an approach could result in greater durability than has been observed with current CSP-based vaccines.

## Materials and methods

### Peptides and antibodies

Seven biotinylated PfCSP peptides spanning across the CSP molecule were produced (CS Bio, Menlo Park, CA), Atlantic Peptides Inc, Concord, NH)) and sequences are listed in **Table 1**. Human monoclonal antibodies (mAb 5D5, CIS43, L9, 317, 236, and 369) were tagged using the MSD Gold-Sulfo-Tag NHS- Ester Conjugation Pack (MesoScale Discovery (MSD) Inc, Gaithersburg, MD), per manufacturer’s instructions. The Sulfo-tagged mAbs were used as competitors, i.e., determining the strength of serum antibodies to compete with the epitope-specific mAbs. The fine specificities of these mAbs are listed in **Table 1**.

**Table 1:** Sequences of peptides and specificity of human mAbs used for the ECLIA-based CBASQE assay.

MSD Spot #	Plate antigen	Sequence	Epitope specific mAb
1	N-terminus	GSSSNTRVLNELNYDNAGTNLYNELEMNYYGKQENWYSLKKNSRSLGENDDGNNEDNEKLRKPKHKKLKQPADG	5D5
2	Junction	KQPADGNPDPNANPNVDP	CIS43
3	Minor repeats	NVDPNANPNVDPNANPNVDPNANP	L9
8	Major repeats	(NPNA)_6_	317
9	C-term 3D7	HNMPNDPNRNVDENANANSAVKNNNNEEPSDKHIKEYLNKIQNSLSTEWSPCSVTCGNGIQVRIKPGSANKPKDELDYANDIEKKICKMEKCS	236, 369
10	C-term H18	mid-Hamming distance = 5 HNMPNDPNRNVDENANANNAVKNNNNEEPSDKHIKEYLNKIQNSISTEWSPCSVTCGNGIQVRIKPGSADKPKDQLDYINDIEKKICKMEKCS	369
7	C-term H50	most divergent from 3D7: Hamming distance = 10 HNMPNDPNRNVDENANANNAVKNNNNEEPSDKHIEQYLKTIKNSLSTEWSPCSVTCGNGIQVRIKPGSAGKSKNELDYENDIEKKICKMEKCS	369

### Clinical test samples

The use of serum samples was reviewed by the WRAIR Human Subjects Protection Branch (Protocol WRAIR#2142). Pre-immune and day of challenge sera (two weeks post third immunization from a previously conducted clinical trial (NCT00075049) (42) in which study participants vaccinated with RTS,S adjuvanted with AS01_B_ or AS02_A_ (n =18 protected subjects, n =18 non-protected subjects) were tested. The study enrolled both, male and female healthy U.S. residents ages 18-45 years with no travel history to malaria-endemic countries. Preliminary experiments did not show differences between the two adjuvant cohorts in the level of their antibody responses to the variant peptides (13) nor their avidity to repeat and C-terminal 3D7 peptide. A human CSP-immune serum pool (CSP-AV) and commercial human AB pooled serum were used as positive and negative assay controls, respectively.

### CBASQE assay

**Figure 1:**
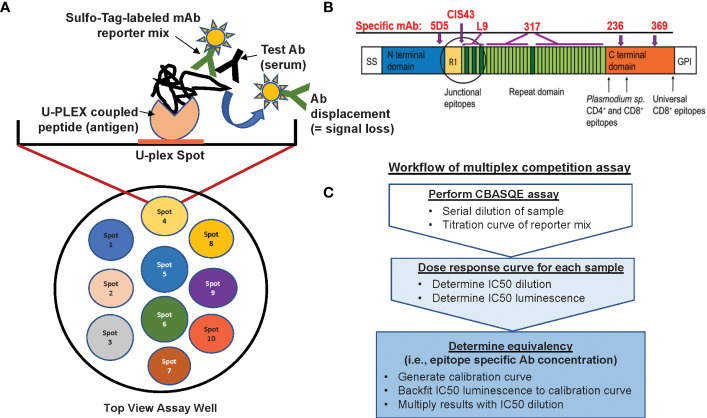
Overview of the CBASQE assay. **(A)** The 10-spot MSD plate accommodates up to ten different antigens per well (left column). Wells were coated with a mixture containing seven U-plex coupled peptides (spots 4-6 were not used). Serially diluted test serum was mixed with the Sulfo-tag labeled mAb reporter mix and added to the U-plex plates. Loss/reduction in luminescence is seen when serum antibodies successfully competed off the Sulfo-tag labeled reporter mix. **(B)** Schematic of the CSP protein visualizing the regions represented by the peptides used in this study and where epitope-specific mAbs are binding. **(C)** Analysis workflow for conducting the serological equivalence assay.

The CBASQE assay is based on the Mesoscale platform and uses the 10-spot, U-PLEX format (MSD, Gaithersburg, MD) (Bolton et al, manuscript in preparation, graphic summary of assay in **Figure 1**). The U-plex format utilizes specific U-plex Linkers for targeted coating of specific spots within the wells of a U-plex plate. Previous work from our lab established that none of the ten available Linkers differ in their efficiency to bind to their specific spot (13).

To prepare the assay plates, each biotinylated peptide (**Table 1**), representing the N-terminus, junctional region, minor repeat, major repeat, and C-terminus, was diluted to 300 nM with the coating diluent (PBS with 0.5% BSA) based on our previous work (13). The peptides were then mixed and incubated at RT with their designated U-plex Linker provided in the MSD U-plex assay kit for 30 mins. MSD Stop Solution (MSD) was added to the peptides/Linker solution (U-PLEX coupled solution) and incubated at RT for another 30 min. After incubation, the individual U-PLEX-coupled solutions were combined into one tube, creating a multiplex coating solution. The 10-spot U-plex plates were then coated with the multiplex coating solution containing the various CSP peptides and incubated at RT for 1hr. After incubation, the U-plex plates were washed with 1x MSD Wash Buffer and used immediately.

During the incubation of the U-PLEX multiplex coating solution, samples were prepared. The reference sample was created by combining unlabeled (no Sulfo-tag) mAb containing, 4.5 ng/mL N-terminus specific mAb 5D5, 1.6 ng/mL junctional peptide-specific mAb CIS43, 14 ng/mL minor repeat-specific mAb L9, 14 ng/mL NPNA_6_-specific mAb 317, and 4.6 ng/mL mAb236 and 4.6 ng/mL mAb 369 for C-term mAbs. This reference sample was tested at eight, 3-fold dilutions (from neat-1:2,187). Test sera were diluted three-fold spanning four dilutions from 1:3 to 1:81.

In a 96 well plate (Corning, Glendale, AZ), the sera samples and the reference sample were combined with the Sulfo-tag reporter mix containing 25 ng/mL mAb 5D5, 25 ng/mL mAb CIS43, 60 ng/mL mAb L9, 25ng/mL mAb 317, 16 ng/mL mAb 236, and 32ng/mL mAb 369 at a constant concentration, then transferred to the antigen-coated U-plex plate, and incubated at RT for 1hr.

After incubation, the plates were washed three times with 1x MSD Wash Buffer. MSD Read Buffer B was added to each well and the plates were immediately read on the MESO QuickPlex SQ 120 (MSD), per the manufacturer’s instructions. Raw data are reported as mean luminescence signal (MLS).

### IC_50_ determination and quantitating the concentration of epitope-specific antibodies

Test sera were diluted three-fold (1:3 to 1:81) to generate a dose response curve based on the percentage inhibition. The dose response curve of each sample is entered into the Quest Graph™ IC50 Calculator (https://www.aatbio.com/tools/ic50-calculator). This calculator models an experimental data set based on a four-parameter logistic regression model and provides the dilution and the luminescence signal corresponding to the IC_50_. Testing eight different concentrations of the reference/reporter mix in each experiment was done to generate a standard curve for (a) the conversion of the IC_50_ into absolute concentrations of epitope-specific antibodies in the sample, and (b) the monitoring of the assay performance from experiment to experiment. The computational manipulations (*i.e*., generating the standard curve and calculating the antibody concentrations) were performed using the Standard Curve Analysis app (OriginLab Inc, Northampton, MA). For the conversion of the IC_50_ to a concentration of epitope-specific serum antibodies, the luminescence signal is backfitted to the standard curve. The calculated IC_50_ concentration is then multiplied with the dilution factor to determine the absolute concentration of epitope-specific antibody (equivalency) in the sample.

### Statistical analysis

Univariate analysis between protected and unprotected subjects was performed to determine the correlation between MSD intensity (reactivity) to the various peptides and protection status. Prior to any t-test, we carried out a Shapiro-Wilks test to determine if the to-be-compared data points were normally distributed. If both were normally distributed (p < 0.05 by the Shapiro-Wilks test), we applied a two-sided Student’s t-test. If either distribution was not normally distributed, we applied the Wilcoxon signed-rank test. Correlations and correlation matrices (to determine the relationship between the various epitope-specificities) were calculated and plotted using R Studio (Version 2023.6.1 + 524). R scripts are available upon request.

## Results

### Serological profiles are significantly different between protected and non-protected individuals

**Figure 2:**
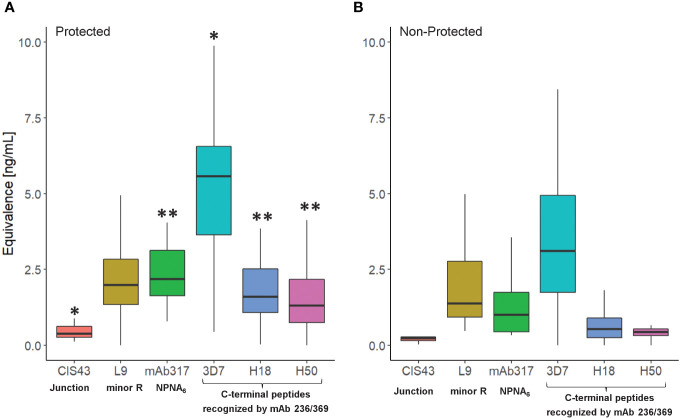
Serological profile of RTS,S vaccinees (NCT00075049) as determined in the CBASQE assay. Data expressed as ng/ml epitope-specific antibody concentration in sera of protected (n=18, **(A)**) or non-protected (n=18, **(B)**) RTS,S vaccinees. Equivalency was determined using mAbs CIS43, 317, 236, and 369. Results for N-terminus omitted since no reactivity of the sera was measured. Asterisks indicate statistical significance between protected *vs.* non-protected RTS,S vaccinees (two-sample T-test, *p< 0.05, **< 0.005).

Establishing the serological profile of malaria-naïve, RTS,S-vaccinated subjects and stratifying the results based on protective status revealed unique roles of repeat- and C-terminal antibodies in protection (**Figure 2**). Based on the vaccine design, we assume that the responses to the junctional sequences are due to the presence of NANP/NVDP in the junctional peptide (KQPADGNPDPNANPNVDP). Interestingly, the magnitude in reactivity to the minor repeat peptide was not statistically different between protected *vs*. non-protected individuals. In contrast, protected individuals had significantly stronger major repeat-specific (NPNA)_6_ and C-terminal antibody responses compared to non-protected individuals. Also noteworthy was the fact that C-terminal antibodies of protected vaccinees demonstrated more cross-reactivity to the H18 and H50 peptides, which agrees with prior observations (15). No responses to the N-terminus were detected confirming the specificity of the assay results since this region is not included in the RTS,S vaccine.

**Figure 3:**
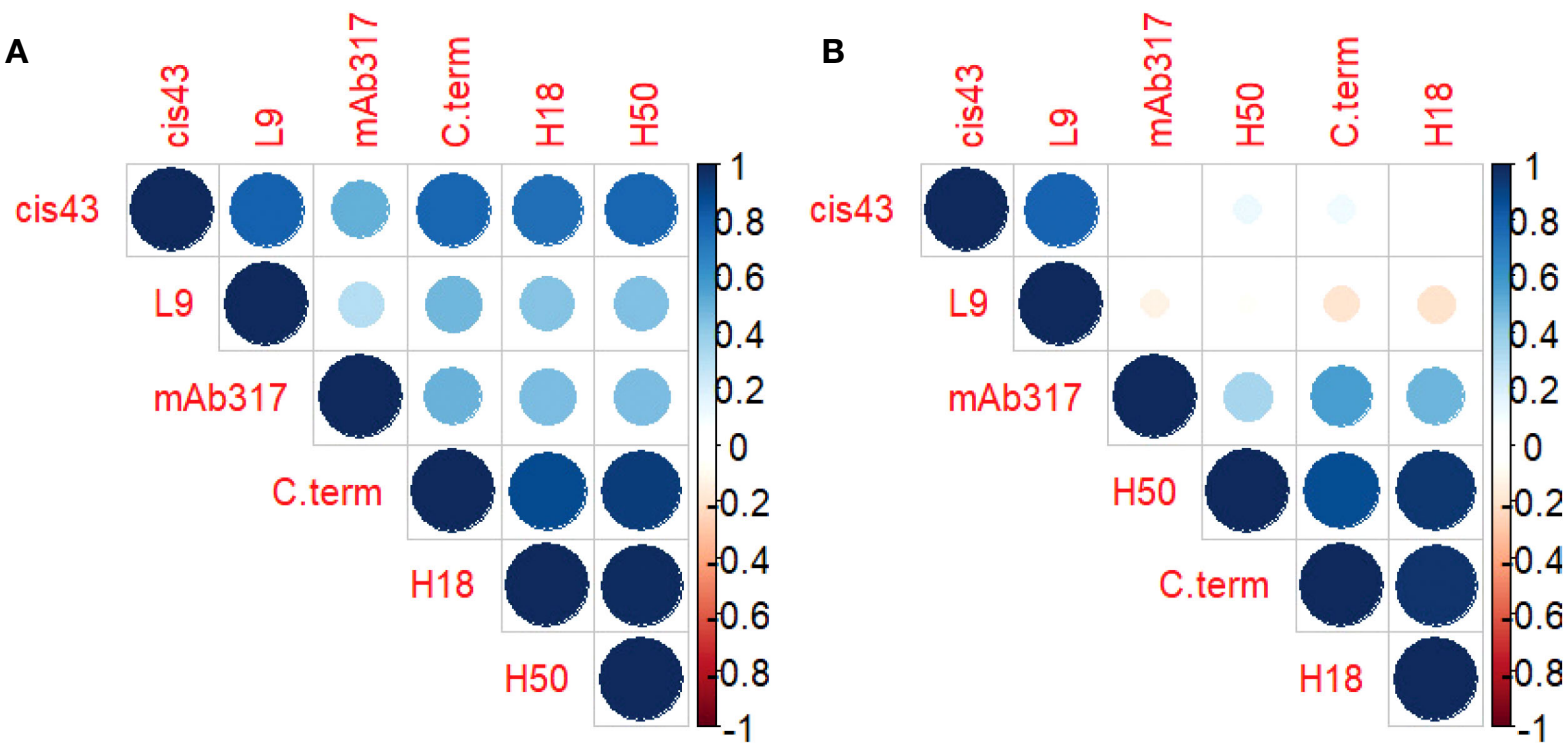
Antibody profile of protected *vs*. non-protected RTS,S immunized vaccinees to CSP regions. Correlation matrices indicate the relationship between the equivalencies of the antibody responses to the various CSP epitope specific mAbs. The color and size of the dots (scale below graphs) indicate the degree of correlation between the different CSP peptides (small to large indicating low to high correlation). Correlation matrices stratified by protective status (**(A)** = protected, **(B)** = non-protected individuals).

Correlation matrices were generated to explore the interplay between the concentrations of epitope-specific antibodies in sera of protected *vs*. non-protected RTS,S vaccinees (**Figure 3**). In protected individuals, significant correlations were observed between the C-terminal specific antibodies (3D7, H18, H50) (**Figure 3A**). Weaker correlations were observed for repeat-specific antibodies with only mAbs L9 and CIS43 correlating significantly. Notably, the magnitude of responses to the C-terminal peptides correlated strongly with the magnitude to the junctional region (CIS43 epitope). Taken together, the data suggest that a broad immune response to various epitopes on CSP is associated with protection. One contributing factor to the stronger correlations in protected individuals can also be a higher quality, *i.e.*, antibodies with higher affinity and/or avidity. High quality antibodies will be more successful in competing with the reporter mAbs than lower quality antibodies. In contrast, the breadth of responses to the various epitopes on CSP in non-protected individuals is limited (**Figure 3B**) to correlations between the C-terminal responses as well as between mAbs CIS43 and L9-epitope specific responses.

### Agreement between ELISA and CBASQE results

Next, we compared the CBASQE data with previously generated ELISA data assessing total IgG, using the same plate antigens for human RTS,S vaccinees (42) to determine the level of agreement between the readout methods. First, we determined the correlation between ELISA and CBASQE on the entire sample set stratified by protective status (**Table 2**).

**Table 2:** Correlation between ELISA and CBASQE results.

	NPNA_6_	C-term (3D7)	H18	H50
All subjects	0.75	0.73	0.85	0.83
Protected	0.76	0.76	0.94	0.91
Non-protected	0.52	0.48	0.36	0.40

**Figure 4:**
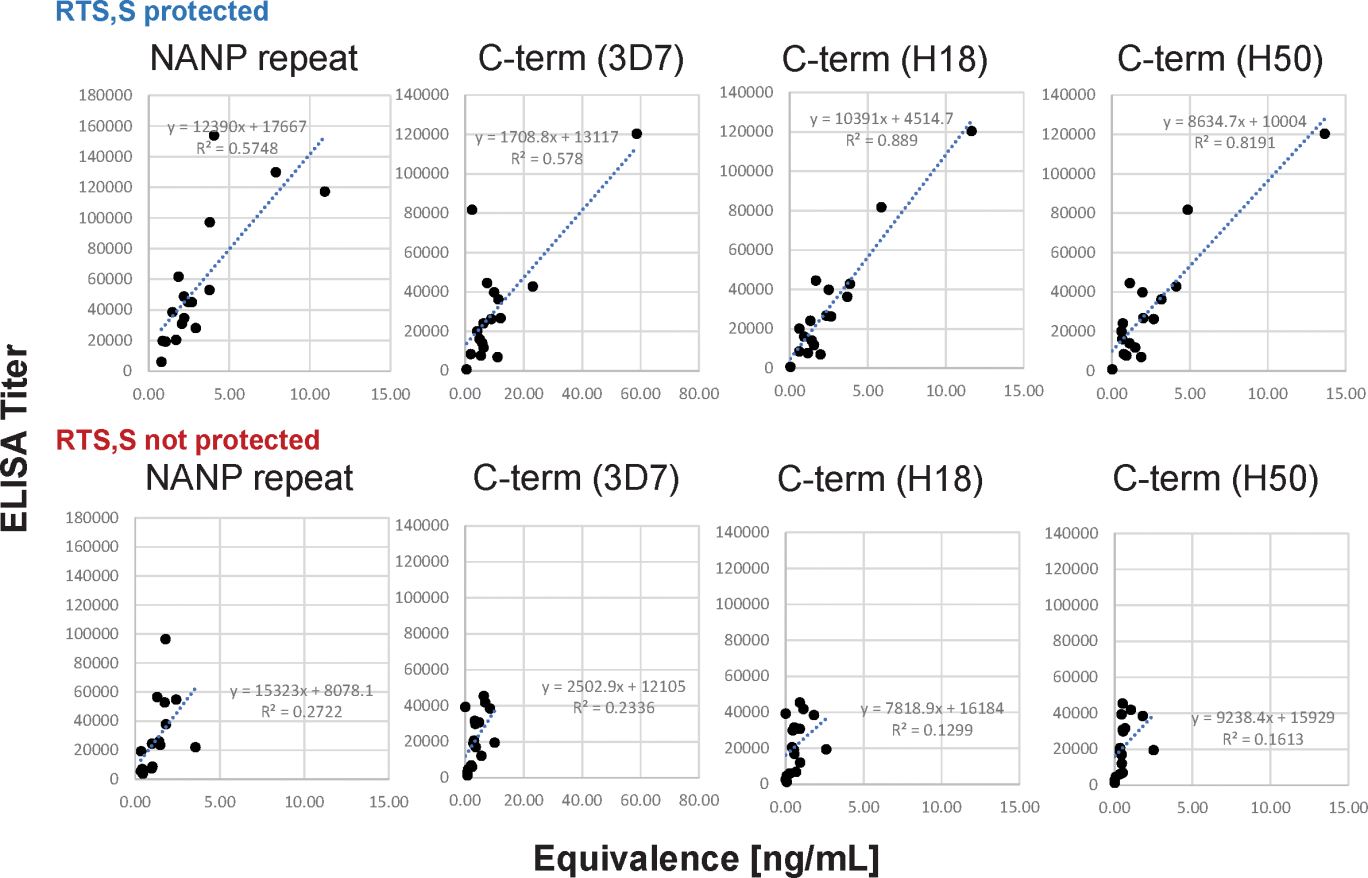
Agreement between ELISA- and CBASQE (Equivalence) results dependent on protective status. Log-transformed ELISA titers (y-axis) and Equivalence (x-axis) results for repeat-specific and C-terminal responses were stratified by protective status: responses of sera from protected (n=18, top panels) and non-protected individuals (n=18, bottom panels).

Overall, the correlations between the data sets are strong when analyzing the data of all subjects (r ≥ 0.73) even though the CBASQE assay detects only selected epitopes across the CSP, i*.e.*, epitopes of the respective mAbs used in the CBASQE assay. Stratifying the data based on protective status, however, revealed there is a qualitative difference between the serological profiles of protected *vs.* non-protected individuals. The degree of correlation was lower in non-protected individuals for both the repeat and the C-terminal region. This difference was visualized by generating scatterplots for each of the plate antigens (**Figure 4**).

## Discussion

Despite decades of rational vaccine design for CSP-based candidates, there is still a major knowledge gap when it comes to immune mechanisms mediating protection against *P. falciparum* infection whether from naturally acquired or vaccine-induced immunity. Sporozoite CSP has been extensively studied as a major target of vaccine development but there is much still unknown regarding critical epitopes of the CSP molecule leading to highly active, durable vaccine responses. The newly developed CBASQE assay simultaneously establishes a serological profile of antigen specificities across the CSP vaccine antigen and measures the quality of the response by determining the concentration of antibodies with equivalence to functionally-active monoclonal antibodies. The CBASQE assay seeks to narrow this knowledge gap and inform next generation vaccine design. Our data demonstrate the following capabilities of the assay:

(a) Sensitive and highly robust assay platform enables the assessment of small volume samples: The high-throughput and high-dimensional assessment of sera is conducive to testing samples from large clinical trials which provide statistical sample sizes adequate for determining the functional role of specific epitopes.

(b) Establishing functional serological profiles of clinical samples (**Figures 2**, **3**): This is invaluable as immunodominance may be different across species and may have an impact on the ability to induce antibodies that have functional activity either *in vitro* or *in vivo*. Conventional serological assays such as ELISA and bead-based flow cytometry (*e.g*., Luminex) used to determine the quality and quantity of antibodies rely on species-specific secondary antibodies. Therefore, direct inter-species comparisons are confounded by the use of different, species-specific secondary reagents. This also hampers inter-laboratory comparisons as different reagents such as different lots of the same secondary antibody can affect the overall results.

(c) Superiority of the CBASQE assay over established ELISA-based methods for assessing the magnitude of serological responses by using CSP-monoclonal, repeat-specific mAbs (43): Our novel assay can assess serological responses in a species-independent manner and report the results as mass concentration. The only serological assay reported to date measures the magnitude of antibody responses to the repeat region and also failed to detect correlation with protection (43).

(d) The CBASQE assay measures the absolute concentration of epitope-specific antibodies in serum/plasma samples and simultaneously infers the binding strength (avidity) of these antibodies by competition with potent mAbs: The distinct feature of the CBASQE assay becomes apparent when comparing the data with ELISA results (**Figure 4**). ELISA titers to the central repeat region have been reported as an immune correlate of protection (8, 44). The CBASQE assay shows a significantly higher concentration of major repeat-directed antibodies in protected subjects (**Figure 2**), but antibodies to the minor repeats were not significantly higher in that cohort. The correlation between ELISA and CBASQE results is strong in protected individuals (r ≥ 0.74) but lower for non-protected individuals suggesting more qualitative differences in the serological response. Qualitative differences between protected and non-protected vaccinees become even more apparent when comparing ELISA *vs*. CBASQE results for C-terminal antibody responses as further discussed below and when comparing the profile (ie., when establishing the breadth of the response to epitopes across the entire CSP molecule) (**Figure 2**).

The overall agreement between ELISA and CBASQE results is only observed in protected RTS,S vaccinees (**Table 2**; **Figure 4**). One possible explanation is the impact of avidity on the CBASQE results. The assay measures epitope-specific antibodies that are capable of successfully competing with specific mAbs. Successful competition of test sera with the epitope specific mAbs is a function of antibody concentration and avidity/affinity. Low-avidity antibodies may not be able to compete with the high-affinity/avidity mAbs in the CBASQE assay but will generate titers in an ELISA assay. Previous work on data integration of serological parameters revealed antibody avidity as one of the hallmarks of a protective response (9).

One of the key differences between ELISA and CASQE assay is the need by ELISAs for secondary reagents that can bias the readout due to their isotype- and subclass-specificity. The CBASQE assay is isotype/subclass agnostic and, therefore, provides a global assessment of the serological response.

We would like to note that the CBASQE assay reports lower concentrations of antigen-specific antibodies in sera compared to quantitative ELISAs. This difference is the result of several factors: (i) most plate antigens present more than one epitope. For example, using the C-terminal peptide Pf16 (45) in ELISA assays results in the binding of a multitude of different antibody clones. In the case of the CBASQE assay, only the binding to mAb 236- and mAb 369-specific epitopes is measured. Therefore, reporting the data as such and distinguishing between a multi-epitope assessment (ELISA) *vs.* a mono- or oligo-epitope assessment (CBASQE) is imperative. Measuring epitope-specific responses rather than the global response to a plate antigen may reveal clear associations between epitope and protection. Another noteworthy aspect is the fact that CBASQE results do not only reflect the magnitude of the epitope specific response, but also include a qualitative assessment (avidity/affinity high enough to compete with the reference mAb).

This present study is the first of its kind in that it applies a competition multiplex assay to assess equivalence of serological responses to known benchmark mAbs (*i.e*., mAbs shown to target critical epitopes and/or mediate functional activity against the pathogen). Here, we used six mAbs specific for distinct epitopes across the CSP molecule. The limited number of mAbs could be seen as a limitation of the assay. When and if new epitopes involved in protection or immune escape are identified (*e.g.*, Friedman-Klabanoff et al, manuscript in preparation), both the peptides representing the respective epitopes and the mAbs can be readily added to the assay. Another limitation of any serological assay seeking to determine correlation of the antibody responses with protection is the fact that the contribution of cellular responses to protection are de-emphasized, apart from the impact of helper T cells on the isotype profile of the antibody response.

The present study addresses the controversial role of antibodies to the CSP’s C-term by revealing that vaccine-induced C-term responses are associated with protection. We had previously reported on a multiplex assay for determining the breadth of C-terminal antibody responses (15). All of the samples for that study were obtained from malaria-naïve RTS,S vaccinees, and the cross-reactivity to other C-terminal variant peptides, defined as the breadth, correlated with protection. In that study, we concluded that the breadth of C-term binding is a surrogate marker for reactivity to conserved epitopes within the C-terminus. Here, we confirm, using a complementary approach, that breadth as measured by the concentration of antibodies that can cross-react with a subset of C-term variants represented by H18 and H50 is also associated with protection (**Figures 2**, **3**). We acknowledge that this assessment was made with the same set of clinical samples. Future studies are planned to assess the generalizability of this observation in other RTS,S as well as other CSP-based vaccine candidate trials.

The objective of this study was to establish a new assay platform that addresses shortcomings of commonly-used readout methods for establishing serological profiles. We used a sample set from an RTS,S clinical trial where participants were challenged (CHMI) after immunization to validate the CBASQE approach. The next step is to apply this new assay to additional, larger CHMI sample sets to further support these findings, and to monitor the development and maturation of protective responses during the course of vaccination. Ultimately, the CBASQE assay will need to be applied to field samples to establish functional serological profiles in residents of malaria-endemic regions (46, 47) as compared to profiles induced by vaccination of malaria-naïve individuals and with various vaccine formulations.

Lastly, this concept of measuring the equivalence of antibody responses in comparison to mAbs with known therapeutic/protective activity or even sera from protected/immune individuals provides a high-throughput readout to decipher functionally relevant serological responses responsible for protection. These data are invaluable for next-generation vaccine design and down-selection of vaccine formulations.

## Data availability statement

The original contributions presented in the study are included in the article/supplementary material. Further inquiries can be directed to the corresponding author.

## Ethics statement

The studies involving humans were approved by WRAIR internal review board/human subject protection branch reviewed and approved the original trial as well as this exempt human use protocol governing this study. The studies were conducted in accordance with the local legislation and institutional requirements. The human samples used in this study were acquired from primarily isolated as part of your previous study for which ethical approval was obtained. Written informed consent for participation was not required from the participants or the participants’ legal guardians/next of kin in accordance with the national legislation and institutional requirements.

## Author contributions

JB: Methodology, Writing – original draft, Investigation, Validation. RM: Investigation, Resources, Writing – review & editing, Visualization. EL: Resources, Writing – review & editing, Investigation, Visualization. JR: Resources, Writing – review & editing, Funding acquisition, Investigation. EB-L: Funding acquisition, Resources, Writing – review & editing, Conceptualization, Data curation, Formal Analysis, Methodology, Visualization, Writing – original draft, Supervision.

## Author disclaimer

Material has been reviewed by the Walter Reed Army Institute of Research. There is no objection to its presentation and/or publication. The opinions or assertions contained herein are the private views of the authors, and are not to be construed as official, or as reflecting the views of the Department of the Army or the Department of Defense. The investigators have adhered to the policies for protection of human subjects as prescribed in AR 70-25. This paper has been approved for public release with unlimited distribution.

## References

[1] Plotkin SA. Updates on immunologic correlates of vaccine-induced protection. Vaccine (2020) 38(9):2250–7. doi: 10.1016/j.vaccine.2019.10.046 31767462

[2] Kelleher K Drysdale C. Who Recommends Groundbreaking Malaria Vaccine for Children at Risk (2019). World Health Organization. Available at: https://www.who.int/news/item/06-10-2021 (Accessed March 16, 2023).

[3] Chandramohan D Zongo I Sagara I Cairns M Yerbanga RS Diarra M. Seasonal malaria vaccination with or without seasonal malaria chemoprevention. New Engl J Med (2021) 385(11):1005–17. doi: 10.1056/NEJMoa2026330 34432975

[4] RTS SCTP. Efficacy and safety of rts,S/as01 malaria vaccine with or without a booster dose in infants and children in Africa: final results of a phase 3, individually randomised, controlled trial. Lancet (2015) 386(9988):31–45. doi: 10.1016/s0140-6736(15)60721-8 25913272 PMC5626001

[5] Cairns M Barry A Zongo I Sagara I Yerbanga SR Diarra M. The duration of protection against clinical malaria provided by the combination of seasonal rts,S/as01(E) vaccination and seasonal malaria chemoprevention versus either intervention given alone. BMC Med (2022) 20(1):352. doi: 10.1186/s12916-022-02536-5 36203149 PMC9540742

[6] WHO. World malaria report, 2022. Geneva/Switzerland: WHO (2022). Available at: https://www.who.int/teams/global-malaria-programme/reports/world-malaria-report-2022.

[7] Datoo MS Natama MH Somé A Traoré O Rouamba T Bellamy D. Efficacy of a low-dose candidate malaria vaccine, R21 in adjuvant matrix-M, with seasonal administration to children in Burkina Faso: A randomised controlled trial. Lancet (2021) 397(10287):1809–18. doi: 10.1016/s0140-6736(21)00943-0 PMC8121760 33964223

[8] Chaudhury S Ockenhouse CF Regules JA Dutta S Wallqvist A Jongert E. The biological function of antibodies induced by the rts,S/as01 malaria vaccine candidate is determined by their fine specificity. Malar J (2016) 15:301. doi: 10.1186/s12936-016-1348-9 27245446 PMC4886414

[9] Chaudhury S Regules JA Darko CA Dutta S Wallqvist A Waters NC. Delayed fractional dose regimen of the rts,S/as01 malaria vaccine candidate enhances an igg4 response that inhibits serum opsonophagocytosis. Sci Rep (2017) 7(1):7998. doi: 10.1038/s41598-017-08526-5 28801554 PMC5554171

[10] Regules JA Cicatelli SB Bennett JW Paolino KM Twomey PS Moon JE. Fractional third and fourth dose of rts,S/as01 malaria candidate vaccine: A phase 2a controlled human malaria parasite infection and immunogenicity study. J Infect Dis (2016) 214(5):762–71. doi: 10.1093/infdis/jiw237 27296848

[11] Young WC Carpp LN Chaudhury S Regules JA Bergmann-Leitner ES Ockenhouse C. Comprehensive data integration approach to assess immune responses and correlates of rts,S/as01-mediated protection from malaria infection in controlled human malaria infection trials. Front Big Data (2021) 4:672460. doi: 10.3389/fdata.2021.672460 34212134 PMC8239149

[12] Bolton JS Chaudhury S Dutta S Gregory S Locke E Pierson T. Comparison of elisa with electro-chemiluminescence technology for the qualitative and quantitative assessment of serological responses to vaccination. Malar J (2020) 19(1):159. doi: 10.1186/s12936-020-03225-5 32303235 PMC7165447

[13] Bolton J Chaudhury S MacGill RS Early AM King CR Locke E. Multiplex serological assay for establishing serological profiles of polymorphic, closely related peptide antigens. MethodsX (2021) 8:101345. doi: 10.1016/j.mex.2021.101345 34430249 PMC8374401

[14] Chaudhury S Bolton JS Eller LA Robb M Ake J Ngauy V. Assessing Prevalence and Transmission Rates of Malaria through Simultaneous Profiling of Antibody Responses against Plasmodium and Anopheles Antigens. J Clin Med (2022) 11(7):1839. doi: 10.3390/jcm11071839 35407447 PMC9000160

[15] Chaudhury S MacGill RS Early AM Bolton JS King CR Locke E. Breadth of humoral immune responses to the C-terminus of the circumsporozoite protein is associated with protective efficacy induced by the rts,S malaria vaccine. Vaccine (2021) 39(6):968–75. doi: 10.1016/j.vaccine.2020.12.055 33431225

[16] Beutler N Pholcharee T Oyen D Flores-Garcia Y MacGill RS Garcia E. A novel CSP C-terminal epitope targeted by an antibody with protective activity against *plasmodium falciparum* . PloS Pathog (2022) 18(3):e1010409. doi: 10.1371/journal.ppat.1010409 35344575 PMC8989322

[17] Thai E Costa G Weyrich A Murugan R Oyen D Flores-Garcia Y. A high-affinity antibody against the csp N-terminal domain lacks plasmodium falciparum inhibitory activity. J Exp Med (2020) 217(11):e20200061. doi: 10.1084/jem.20200061 32790871 PMC7596816

[18] Wang LT Pereira LS Kiyuka PK Schön A Kisalu NK Vistein R. Protective effects of combining monoclonal antibodies and vaccines against the *plasmodium falciparum* circumsporozoite protein. PloS Pathog (2021) 17(12):e1010133. doi: 10.1371/journal.ppat.1010133 34871332 PMC8675929

[19] Pholcharee T Oyen D Torres JL Flores-Garcia Y Martin GM González-Páez GE. Diverse antibody responses to conserved structural motifs in *plasmodium falciparum* circumsporozoite protein. J Mol Biol (2020) 432(4):1048–63. doi: 10.1016/j.jmb.2019.12.029 PMC7057269 31883801

[20] Flores-Garcia Y Wang LT Park M Asady B Idris AH Kisalu NK. The P. Falciparum CSP repeat region contains three distinct epitopes required for protection by antibodies *in vivo* . PloS Pathog (2021) 17(11):e1010042. doi: 10.1371/journal.ppat.1010042 34748617 PMC8601602

[21] Wang LT Pereira LS Flores-Garcia Y O’Connor J Flynn BJ Schön A. A potent anti-malarial human monoclonal antibody targets circumsporozoite protein minor repeats and neutralizes sporozoites in the liver. Immunity (2020) 53(4):733–44.e8. doi: 10.1016/j.immuni.2020.08.014 32946741 PMC7572793

[22] Pholcharee T Oyen D Flores-Garcia Y Gonzalez-Paez G Han Z Williams KL. Structural and biophysical correlation of anti-nanp antibodies with *in vivo* protection against P. Falciparum. Nat Commun (2021) 12(1):1063. doi: 10.1038/s41467-021-21221-4 33594061 PMC7887213

[23] Oyen D Torres JL Wille-Reece U Ockenhouse CF Emerling D Glanville J. Structural basis for antibody recognition of the nanp repeats in *plasmodium falciparum* circumsporozoite protein. Proc Natl Acad Sci (2017) 114(48):E10438–E45. doi: 10.1073/pnas.1715812114 PMC5715787 29138320

[24] Martin GM Torres JL Pholcharee T Oyen D Flores-Garcia Y Gibson G. Affinity-matured homotypic interactions induce spectrum of pfcsp-antibody structures that influence protection from malaria infection. bioRxiv (2022), 2022.09.20.508747. doi: 10.1101/2022.09.20.508747 PMC10382551 37507365

[25] Coppi A Natarajan R Pradel G Bennett BL James ER Roggero MA. The malaria circumsporozoite protein has two functional domains, each with distinct roles as sporozoites journey from mosquito to mammalian host. J Exp Med (2011) 208(2):341–56. doi: 10.1084/jem.20101488 PMC3039851 21262960

[26] Aldrich C Magini A Emiliani C Dottorini T Bistoni F Crisanti A. Roles of the amino terminal region and repeat region of the *plasmodium berghei* circumsporozoite protein in parasite infectivity. PloS One (2012) 7(2):e32524. doi: 10.1371/journal.pone.0032524 22393411 PMC3290588

[27] Gaudinski MR Berkowitz NM Idris AH Coates EE Holman LA Mendoza F. A monoclonal antibody for malaria prevention. New Engl J Med (2021) 385(9):803–14. doi: 10.1056/NEJMoa2034031 PMC8579034 34379916

[28] Wu RL Idris AH Berkowitz NM Happe M Gaudinski MR Buettner C. Low-dose subcutaneous or intravenous monoclonal antibody to prevent malaria. New Engl J Med (2022) 387(5):397–407. doi: 10.1056/NEJMoa2203067 35921449 PMC9806693

[29] Oyen D Torres JL Cottrell CA Richter King C Wilson IA Ward AB. Cryo-em structure of P. Falciparum circumsporozoite protein with a vaccine-elicited antibody is stabilized by somatically mutated inter-fab contacts. Sci Adv (2018) 4(10):eaau8529. doi: 10.1126/sciadv.aau8529 30324137 PMC6179375

[30] Pholcharee T Oyen D Flores-Garcia Y Gonzalez-Paez G Han Z Williams KL. Structural and biophysical correlation of anti-nanp antibodies with *in vivo* protection against P. Falciparum. Nat Commun (2021) 12(1):1063. doi: 10.1038/s41467-021-21221-4 33594061 PMC7887213

[31] Murugan R Scally SW Costa G Mustafa G Thai E Decker T. Evolution of protective human antibodies against plasmodium falciparum circumsporozoite protein repeat motifs. Nat Med (2020) 26(7):1135–45. doi: 10.1038/s41591-020-0881-9 32451496

[32] Imkeller K Scally SW Bosch A Martí GP Costa G Triller G. Antihomotypic affinity maturation improves human B cell responses against a repetitive epitope. Science (2018) 360(6395):1358–62. doi: 10.1126/science.aar5304 PMC6420115 29880723

[33] Kisalu NK Idris AH Weidle C Flores-Garcia Y Flynn BJ Sack BK. A human monoclonal antibody prevents malaria infection by targeting a new site of vulnerability on the parasite. Nat Med (2018) 24(4):408–16. doi: 10.1038/nm.4512 PMC5893371 29554083

[34] Neafsey DE Juraska M Bedford T Benkeser D Valim C Griggs A. Genetic diversity and protective efficacy of the rts,S/as01 malaria vaccine. New Engl J Med (2015) 373(21):2025–37. doi: 10.1056/NEJMoa1505819 PMC4762279 26488565

[35] Scally SW Murugan R Bosch A Triller G Costa G Mordmuller B. Rare pfcsp C-terminal antibodies induced by live sporozoite vaccination are ineffective against malaria infection. J Exp Med (2018) 215(1):63–75. doi: 10.1084/jem.20170869 29167197 PMC5748854

[36] Ubillos I Ayestaran A Nhabomba AJ Dosoo D Vidal M Jimenez A. Baseline exposure, antibody subclass, and hepatitis B response differentially affect malaria protective immunity following rts,S/as01e vaccination in African children. BMC Med (2018) 16(1):197. doi: 10.1186/s12916-018-1186-4 30376866 PMC6208122

[37] Dobano C Sanz H Sorgho H Dosoo D Mpina M Ubillos I. Concentration and avidity of antibodies to different circumsporozoite epitopes correlate with rts,S/as01e malaria vaccine efficacy. Nat Commun (2019) 10(1):2174. doi: 10.1038/s41467-019-10195-z 31092823 PMC6520358

[38] Dennison SM Reichartz M Abraha M Spreng RL Wille-Reece U Dutta S. Magnitude, specificity, and avidity of sporozoite-specific antibodies associate with protection status and distinguish among rts,S/as01 dose regimens. Open Forum Infect Dis (2020) 8(2). doi: 10.1093/ofid/ofaa644

[39] Suscovich TJ Fallon JK Das J Demas AR Crain J Linde CH. Mapping functional humoral correlates of protection against malaria challenge following rts,S/as01 vaccination. Sci Transl Med (2020) 12(553). doi: 10.1126/scitranslmed.abb4757 32718991

[40] Laurens MB. Rts,S/as01 vaccine (Mosquirix): an overview. Hum Vaccin Immunother (2020) 16(3):480–9. doi: 10.1080/21645515.2019.1669415 PMC7227679 31545128

[41] Collins KA Snaith R Cottingham MG Gilbert SC Hill AVS. Enhancing protective immunity to malaria with a highly immunogenic virus-like particle vaccine. Sci Rep (2017) 7:46621. doi: 10.1038/srep46621 28422178 PMC5395940

[42] Kester KE Cummings JF Ofori-Anyinam O Ockenhouse CF Krzych U Morris P. Randomized, double-blind, phase 2a trial of falciparum malaria vaccines rts,S/as01b and rts,S/as02a in malaria-naive adults: safety, efficacy, and immunologic associates of protection. J Infect Dis (2009) 200:337–46. doi: 10.1086/600120 19569965

[43] Radin K Clement F Jongert E Sterckx YGJ Ockenhouse C Regules J. A monoclonal antibody-based immunoassay to measure the antibody response against the repeat region of the circumsporozoite protein of *plasmodium falciparum* . Malaria J (2016) 15(1):543. doi: 10.1186/s12936-016-1596-8 PMC5101676 27825382

[44] White MT Bejon P Olotu A Griffin JT Riley EM Kester KE. The relationship between rts,S vaccine-induced antibodies, cd4(+) T cell responses and protection against *plasmodium falciparum* infection. PloS One (2013) 8(4):e61395. doi: 10.1371/journal.pone.0061395 23613845 PMC3628884

[45] Schwenk R DeBot M Porter M Nikki J Rein L Spaccapelo R. Igg2 Antibodies against a Clinical Grade *Plasmodium Falciparum* CSP Vaccine Antigen Associate with Protection against Transgenic Sporozoite Challenge in Mice. PloS One (2014) 9(10):e111020. doi: 10.1371/journal.pone.0111020 25343487 PMC4208815

[46] Triller G Scally SW Costa G Pissarev M Kreschel C Bosch A. Natural parasite exposure induces protective human anti-malarial antibodies. Immunity (2017) 47(6):1197–209.e10. doi: 10.1016/j.immuni.2017.11.007 29195810 PMC5738265

[47] Raghavan M Kalantar KL Duarte E Teyssier N Takahashi S Kung AF. Antibodies to repeat-containing antigens in *plasmodium falciparum* are exposure-dependent and short-lived in children in natural malaria infections. eLife (2023) 12:e81401. doi: 10.7554/eLife.81401 36790168 PMC10005774

